# Maternal Sensitivity Buffers the Association between SLC6A4 Methylation and Socio-Emotional Stress Response in 3-Month-Old Full Term, but not very Preterm Infants

**DOI:** 10.3389/fpsyt.2017.00171

**Published:** 2017-09-13

**Authors:** Livio Provenzi, Monica Fumagalli, Roberto Giorda, Francesco Morandi, Ida Sirgiovanni, Uberto Pozzoli, Fabio Mosca, Renato Borgatti, Rosario Montirosso

**Affiliations:** ^1^0-3 Center for the at-Risk Infant, Scientific Institute IRCCS Eugenio Medea, Lecco, Italy; ^2^NICU, Department of Clinical Sciences and Community Health, Università degli Studi di Milano, Fondazione IRCCS Ca’ Granda Ospedale Maggiore Policlinico, Milan, Italy; ^3^Molecular Biology Laboratory, Scientific Institute IRCCS Eugenio Medea, Lecco, Italy; ^4^Pediatric Unit, Sacra Famiglia Hospital, Como, Italy; ^5^Bioinformatic Lab, Scientific Institute IRCCS Eugenio Medea, Lecco, Italy; ^6^Neuropsychiatry and Neurorehabilitation Unit, Scientific Institute IRCCS Eugenio Medea, Lecco, Italy

**Keywords:** DNA methylation, maternal sensitivity, negative emotionality, next generation sequencing, serotonin transporter gene, *SLC6A4*, stress response, very preterm infants

## Abstract

**Background:**

Very preterm (VPT) infants are hospitalized in Neonatal Intensive Care Units (NICUs) and are exposed to life-saving procedures eliciting pain-related stress. Recent research documented that pain-related stress might result in birth-to-discharge increased methylation of serotonin transporter gene (*SLC6A4*) in VPT infants, leading to poorer stress regulation at 3 months of age in VPT infants compared to their full-term (FT) counterparts. Maternal sensitivity is thought to support infants’ stress response, but its role in moderating the effects of altered *SLC6A4* methylation is unknown.

**Main aim:**

To assess the role of maternal sensitivity in moderating the association between altered *SLC6A4* methylation and stress response in 3-month-old VPT and FT infants.

**Methods:**

53 infants (27 VPTs, 26 FTs) and their mothers were enrolled. *SLC6A4* methylation was obtained from peripheral blood samples at NICU discharge for VPT infants and from cord blood at birth for FT infants. At 3 months (age corrected for prematurity), both groups participated to the face-to-face still-face (FFSF) paradigm to measure both infants’ stress response (i.e., negative emotionality) and maternal sensitivity.

**Results:**

Maternal sensitivity did not significantly differ between VPT and FT infants’ mothers. In VPT infants, higher *SLC6A4* methylation at hospital discharge associates with higher negative emotionality during the FFSF. In FT infants, *SLC6A4* methylation and maternal sensitivity significantly interacted to predict stress response: a positive significant association between *SLC6A4* methylation and negative emotionality emerged only in FT infants of less-sensitive mothers.

**Discussion:**

Although no differences emerged in caregiving behavior in the two groups of mothers, maternal sensitivity was effective in moderating the effects of *SLC6A4* methylation in FT infants, but not in VPT infants at 3 months. Speculatively, the buffering effect of maternal sensitivity observed in FT infants was disrupted by the altered early mother–infant contact due to NICU stay of the VPT group. These findings indirectly support that the effects of maternal sensitivity on infants’ socio-emotional development might be time dependent, and that mother–infant interventions in the NICU need to be provided precociously within a narrow sensitive period after VPT birth.

## Introduction

Very preterm (VPT, gestational age <32 weeks) infants are exposed to numerous stressors in the Neonatal Intensive Care Unit (NICU) (1), including high levels of pain-related stress (i.e., skin-breaking procedures), which can induce long-lasting detrimental effects on subsequent capacities to regulate stress (2, 3). Even when controlling for perinatal and medical confounders, the number of skin-breaking procedures to which they are exposed has been associated with negative later outcomes including less-than-optimal physiological stress response (4) and behavioral response (5). Epigenetic mechanisms refer to the regulation of genes’ transcriptional activity through experience and environmental stimulations and have been invoked as a potential explanation underlying the adverse effects of early NICU-related stress exposure (6). Nonetheless, research on animal models and human subjects suggest that postnatal maternal sensitive behaviors may buffer the early epigenetic effects of adverse experiences on the further development of stress response capacities (7–9). In the current paper, we assessed the association between DNA methylation of a specific stress-related gene, which encodes for serotonin transporter (i.e., *SLC6A4*) and socio-emotional stress response in 3-month-old VPT infants compared to full-term (FT) controls, examining the potential buffering role of maternal sensitivity.

## Background

### The Serotonergic System and Epigenetic Variations

The serotonergic system plays a pivotal role in socio-emotional stress response (10, 11). Serotonin (5-HT) receptors are widely distributed throughout the central nervous system, they appear early during gestation, and the entire serotonergic system rapidly develops during the first months of life (12). The functioning of the serotonergic system is regulated by feedback mechanisms through the serotonin transporter (5-HTT), which is encoded by the *SLC6A4* gene (13). The transcriptional activity of the *SLC6A4* is regulated both by genetic variations and epigenetic mechanisms. Among the genetic variants, previous studies have mainly investigated the role of a transporter-linked polymorphic region (i.e., the 5-HTTLPR polymorphism) in affecting infants’ stress response (14–17). The 5-HTTLPR has a short (S) and long (L) allelic variants (18), with the former being linked to reduced 5-HTT transcription and heightened risk for adverse developmental outcomes, including socio-emotional dysregulation and stress susceptibility (10).

Notably, the genetically determined variability conveyed by the 5-HTTLPR polymorphism has proved to be insufficient to independently account for differences in socio-emotional stress response and susceptibility (19, 20). As such, during the last decade, epigenetic mechanisms regulating *SLC6A4* transcription have been increasingly studied in association with early adversities and behavioral developmental outcomes (21). DNA methylation is the most widely studied epigenetic mechanisms in humans, and it consists in the addition of a methyl group to the specific CpG sites within a gene promoter region, leading to reduced transcriptional activity and, eventually, to gene silencing (22). Converging evidence suggests that increased *SLC6A4* methylation might be a marker of early adversities in humans and might play a role in altered developmental trajectories of stress response and susceptibility (23). For example, maternal depression during pregnancy (24), childhood maltreatment (25), and disadvantaged socioeconomic conditions (26) have been found to associate with CpG-specific patterns of altered methylation within the *SLC6A4* promoter region.

### Infants’ Socio-Emotional Stress Response during the Face-to-Face Still-Face (FFSF) Paradigm

Socio-emotional stress response refers to the ability to respond adaptively to an interpersonal challenging condition (27). Early infants’ socio-emotional stress response is thought to be predictive of adjustment later in life (28). Better stress response has been shown to contribute to adaptive socio-emotional functioning (29), as well as to the development of secure attachment (30). Past research has suggested that infants’ socio-emotional stress response is particularly apparent in response to a brief relational disruption during the FFSF paradigm (31). During the FFSF, infants first engage in normal face-to-face interaction with their caregiver (Play episode). After that, mothers are asked to exhibit a neutral facial expression, avoid touching, and responding to their infant (Still-Face episode). During the Still-Face episode, infants usually exhibit a typical behavioral pattern of stress response (i.e., *still-face* effect), characterized by increased negative emotionality (32). Subsequently, mothers and infants resume their face-to-face interaction as in the first episode (Reunion episode). The FFSF paradigm has been extensively used to study infants’ socio-emotional stress response to maternal unresponsiveness (33, 34).

### Contributors of Infants’ Socio-Emotional Stress Response: Birth Status and Early Maternal Sensitivity

Previous FFSF research documented that infants’ socio-emotional stress response might be affected by both infants [e.g., VPT birth; (35, 36)] and caregiver [e.g., maternal sensitivity; (30, 37)]. A handful of studies have reported poorer regulatory skills in preterm infants compared to their FT counterpart. For example, moderately preterm infants (i.e., gestational age between 31 and 34 weeks) show heightened susceptibility to maternal still-face at 2 months (38), reduced self-soothing attempts at 3 months during the Still-Face episode (39), and more avoiding strategies at 9 months during the Reunion episode (40).

Caregiving behavior is another factor affecting infants’ stress response. The social buffering hypothesis suggests that maternal sensitivity may moderate the effects of early stress exposures on infants’ behavior and physiology (41, 42). Sensitivity is generally defined as a set of maternal caregiving features including the ability to detect infants’ gross and subtle communicative signals, the acceptance of infants’ behavioral signals and requests, the providing of responses contingent in time and intensity to infants’ signals, the mirroring and scaffolding of infants’ socio-emotional experience in tactile and face-to-face interactions. Maternal sensitivity is associated with an increased ability to regulate stress in human infants (43–45), and it is associated with better developmental outcomes later in life (46). Importantly, maternal behavior has been found to mediate the susceptibility effects conveyed by the 5-HTTLPR S allele in 4-month-old infants during the FFFS: high levels of maternal social engagement reduced the negative emotionality exhibited by S-carrier infants to levels comparable to those of L-homozygous subjects (15).

### Epigenetic Contribution to VPT Infants’ Socio-Emotional Stress Response

Behavioral epigenetics has been applied to the study of VPT infants’ socio-emotional development (6). Emerging evidence suggests that epigenetic mechanisms might be involved in setting the risk of altered developmental trajectories of stress response in VPT infants. For example, despite no significant difference in *SLC6A4* methylation was detected at birth between VPT and FT infants, a CpG-specific methylation increase was observed at discharge within the promoter region of the serotonin transporter gene in VPT infants, as a function of the number of skin-breaking procedures to which they were exposed during the NICU hospitalization (47). When these infants were exposed to the FFSF procedure, they showed heightened stress susceptibility compared to FT controls and the amount of negative emotionality exhibited during both Still-Face#2 and Reunion#2 episodes was predicted by the CpG-specific increase in *SLC6A4* methylation observed at NICU discharge (48). In a cross-sectional study, Chau et al. (49) reported a similar association of early pain-related stress exposure in NICU and 7-year-old VPT children, further suggesting that the *SLC6A4* methylation at 7 years was significantly correlated with an increased risk of behavioral problems. Taken together, these findings suggest that epigenetic mechanisms might contribute to long-lasting alterations of the *SLC6A4* methylation status and to associated detrimental effects on behavioral and socio-emotional development during infancy and childhood.

### Maternal Sensitivity As an Epigenetic Regulator

Recent research also highlights that variations in the quality of caregiver behavior has an impact on the epigenetic status of stress-related genes (9). Rodent offspring raised by mothers characterized by low levels of sensitive caregiving exhibited high methylation and reduced expression of the gene encoding for glucocorticoid receptors (i.e., *NR3C1*) and had lower stress response skills during adulthood (50). Nonetheless, when these rodents were cross-fostered to mothers characterized by high levels of care quality, these effects were reversed (9). Although preliminary, similar evidence has recently emerged for humans. Conradt et al. (7) documented that mothers who were more responsive and who engaged in more appropriate touch during face-to-face interactions had infants with less CpG-specific methylation of a stress-related gene (i.e., *NR3C1*) compared to mothers characterized by low sensitivity. This evidence gives new support to the hypothesis that quality of early maternal sensitivity might moderate the effects of early epigenetic alterations due to stressful conditions even in human infants. The potential buffering effect of maternal caregiving on epigenetic changes observed in VPT infants has been recently suggested (51, 52), but there is no evidence to date.

### The Present Study

Previous research documented that NICU-related stress might contribute to altered *SLC6A4* methylation in VPT infants (47) and that these epigenetic variations might lead to increased socio-emotional stress susceptibility at 3 months in response to the FFSF procedure (48). Using the FFSF paradigm, the present study was aimed at exploring the potential buffering role of maternal sensitivity in moderating the relationship between CpG-specific *SLC6A4* methylation and 3-month socio-emotional stress response in VPT and FT infants (corrected age for prematurity, CA). Consistent with the abovementioned literature on maternal buffering effect, we hypothesized that high levels of maternal sensitivity would be associated with better socio-emotional stress response in both groups.

## Methods

### Participants

The present study is part of a longitudinal research project on the epigenetic and behavioral effects of early adverse experiences in NICU in VPT infants. In previous works, we have reported partial data about *SLC6A4* methylation and infants’ behavioral development during the first months of life (47, 48, 53). Here, we focus on the potential role of maternal sensitivity in moderating the association between altered *SLC6A4* methylation and stress response at 3 months CA. A cohort of 88 infants and their mothers was consecutively recruited between October 2011 and April 2014. Of the initial sample, 53 infants had complete infant and maternal data at 3 months CA and were included in the present study. Included and excluded infants did not differ for any of sociodemographic and perinatal variables. VPT infants (*N* = 27, 14 females) were enrolled at the NICU of the Department of Clinical Sciences and Community Health, Fondazione IRCCS Ca’ Granda Ospedale Maggiore Policlinico of Milan (MI, Italy). Inclusion criteria for VPT infants were: gestational age less than 32 weeks and/or birth weight ≤1,500 g, no major brain lesions on cerebral ultrasound (intraventricular hemorrhage >2 according to Papile, periventricular leukomalacia >1), no neuro-sensorial deficits (retinopathy of prematurity >2), no genetic syndromes, and/or major malformations. FT infants (*N* = 26, 14 females) were recruited at the Pediatric Unit of the Sacra Famiglia Hospital, Erba (CO, Italy). FT infants were all healthy, with gestational age ≥37 weeks, no postnatal complications, or prenatal/perinatal risk factors. For both groups, mothers’ inclusion criteria were as follows: Italian nationality, age over 18 years and no single parent. Medical charts were used to screen and exclude mothers documenting cognitive impairments, manifest psychiatric disorders, prenatal depression or anxiety, use of psychotropic medication during pregnancy or drug/alcohol addiction. Mothers and fathers of all participating infants provided written informed consent. According to previous studies on this topic (47), the present sample size appears to be adequate to detect significant variations in CpG-specific methylation of the SLC6A4 gene with alpha error set at 5% and beta error set at 10% (i.e., statistical power = 0.90). The research project has been conducted in accordance with the Code of Ethics of the World Medical Association (Declaration of Helsinki, seventh revision, 2013) and the study has been approved by the Ethics Committees of the Scientific Institute IRCCS Eugenio Medea, Bosisio Parini (LC, Italy) and participating hospitals.

### Procedures

**Figure 1:**
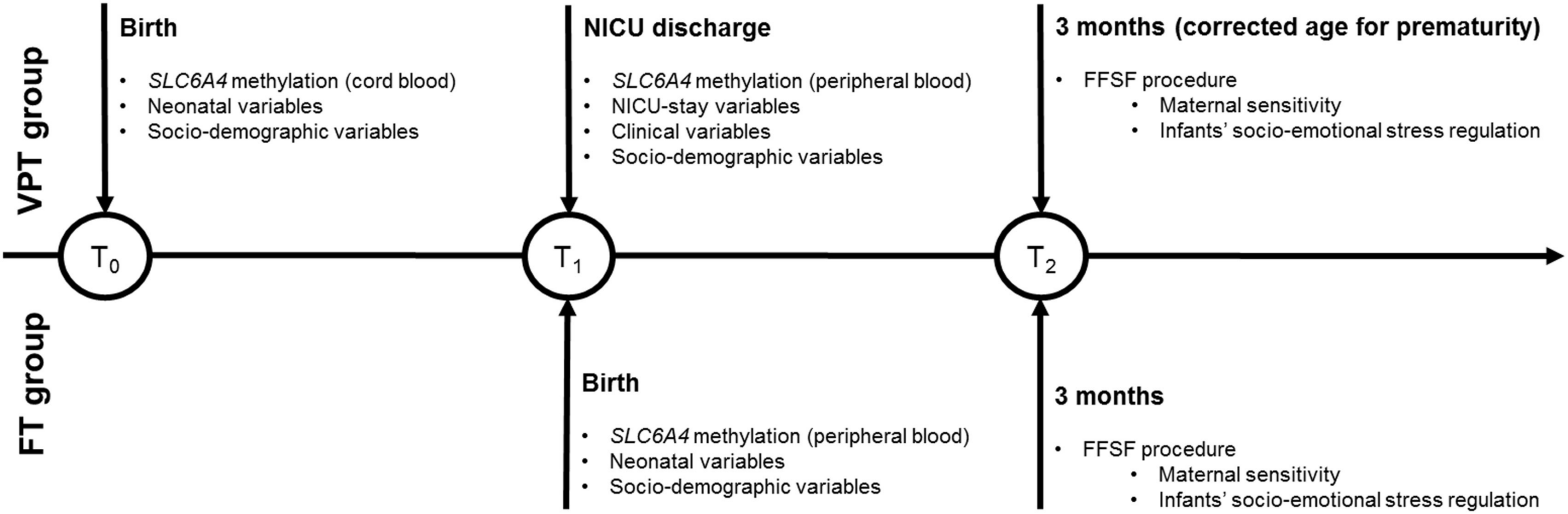
Overview of the PBE longitudinal research project. Note. VPT, very preterm; FT, full-term; NICU, Neonatal Intensive Care Unit; FFSF, Face-to-Face Still-Face.

An overview of the research study plan and the timing of epigenetic and behavioral procedures is reported in Figure 1. The NICU was a traditional open space level III unit, where parents had 24-h access (54). Developmental care was not clustered in protocols (e.g., Newborn Individualized Developmental Care and Assessment program, NIDCAP). The NICU had a maximum of 23 incubators, without single-room facilities. The parents had reclining chairs available throughout the stay. Consistently to previous research (24, 47) and to control for post-conceptional age at the epigenetic assessment, methylation was assessed from cord blood at NICU discharge in VPT infants and from cord blood at birth in FT controls. Blood samples were obtained by trained nurses to avoid hemolysis and immediately stored at −20°C and sent to the lab. Infants’ clinical variables were obtained from medical records. At 3 months CA, the FFSF procedure occurred during a home visit and mothers completed a sociodemographic survey including the collection of neonatal variables and maternal sociodemographic characteristics as well as maternal depressive and anxious symptoms.

#### FFSF Paradigm

The double-exposure FFSF (55, 56) has three face-to-face interactive 2-min episode (Play, Reunion #1, Reunion #2), which are alternate with 2-min Still-Face episodes #1 and #2. During both Still-Face episodes, the mother is instructed to look at the infant keeping a neutral facial expression and avoiding any communicative sign and gesture toward the infant. The double-exposure FFSF procedure has been recently used to assess the stress response of VPT and FT infants to cumulative socio-emotional stress (48). The advantages of using a double-exposure FFSF procedure in research on infants’ socio-emotional stress regulation include: (a) the possibility to assess infants’ response to both acute and repeated exposures to maternal unresponsiveness (56), and (b) the elicitation of a moderate, still robust, and age-appropriate socio-emotional stress in infants so that biological systems activate and precise indexes of stress reactivity can be obtained (27). Two meta-analytic studies documented that the FFSF procedure is a reliable procedure to collect information on socio-emotional stress regulation in FT and preterm infants (33, 34). Moreover, the double-exposure version of the original FFSF procedure has been found to be specifically useful to obtain information on infants’ regulatory behaviors when an interactive condition critically challenges their capacity to cope and react to stress (55). In order to reach the aims of the present study, two cameras were used: one focused on the infant and one on the mother. The mother was approximately 0.4 m from the infant with her eyes leveled at her infant’s eyes. Signals from the two cameras were edited offline to produce a single image for coding purposes.

### Measures

#### Infant Perinatal Data

Perinatal variables that were obtained from medical records included: gestational age, birth weight, gender, Apgar score, length of NICU stay, days on mechanical ventilation (i.e., conventional ventilation and high frequency ventilation)—the last two variables were collected only for VPT infants.

#### Sociodemographic Characteristics

Sociodemographic data such as maternal age, years of study, and occupation were obtained through questionnaires. According to Hollingshead’s classification (57), the more prestigious occupational level between mother and father (i.e., the highest of the two ratings) was considered to indicate the family socioeconomic status (SES). Score ranges from 0 (occupations that do not require high school graduation) to 90 (occupations that require highly specialized education and training).

#### Pain-Related Stress Index

Neonatal pain-related stress was quantified according to previous literature (58, 59). In the present sample, no VPT infants underwent surgery and chest tube insertions. Consequently, pain-related stress was quantified as the total number of skin-breaking procedures throughout the NICU stay, including heel lance, arterial and venous punctures, and peripheral venous line insertion.

#### SLC6A4 Methylation

We analyzed a CpG-rich region of the *SLC6A4* promoter (chr17:28562750.28562958, Human hg19 Assembly), between −69 and −213 relative to the transcriptional start site, which contains 20 CpG sites and is adjacent to exon 1A. Methylation levels were determined in DNA using bisulfite modification followed by PCR amplification and NGS. Genomic DNA was extracted from 0.2 ml of each sample using the GenElute Blood Genomic DNA kit (Sigma). Bisulfite conversion was performed on 500 ng of genomic DNA using the EZ DNA methylation kit (ZymoResearch, Inc., Irvine, CA, USA). Primers were designed using Bisulfite Primer Seeker. The gene-specific forward 5′-GYGGGTTTTTATATGGTTTGATTTTTAG-3′ and reverse 5′-CRAAAATCCCTCCCCTCCTAACTCTAAAATC-3′ primers were sequenced. A TruSeq amplicon-specific tail 5′-CCTACACGACGCTCTTCCGATCT-3′ was added to the forward primer, while the sequence 5′-TCAGACGTGTGCTCAACCGATCT-3′ was added to the reverse primer, in order to allow synthesis and sequencing of TruSeq libraries of methylated fragments. Primary PCR-amplification was performed on 20 ng of bisulfite-treated DNA using Taq Gold (Life Technologies, Inc.). Cycling comprised 5 min preactivation at 95°C, followed by 35 cycles of 94°C denaturation for 15 s, 58°C annealing for 20 s, 72°C elongation for 1.5 min. All PCR products were checked on 2% agarose gel and treated with Ilustra Exo Pro-STAR (GE Healthcare) to eliminate unincorporated primers. Secondary PCR was conducted on each sample using a TruSeq Custom Amplicon Index Kit (Illumina) containing eight forward (i5) and 12 reverse (i7) index primers. Optimal annealing temperature (68°C) and number of PCR cycles were experimentally determined. Cycling comprised 5 min preactivation at 95°C, followed by 16 cycles of 94°C denaturation for 15 s, 68°C annealing for 20 s, 72°C elongation for 1 min. All PCR products were checked on 2% agarose gel, and approximately equimolar aliquots of each product were pooled and purified on a 2% agarose gel. The purified library was quantified on a Bioanalyzer 2100 (Agilent) and sequenced on a MiSeq (Illumina) using a v2 Reagent kit, 300 cycles PE. Paired ends reads from each sample were independently aligned to all the reference sequences by a parallel striped Smith–Waterman algorithm. Only paired reads that aligned coherently to the same reference sequence were retained. At each CpG site in each sequence, the four bases frequencies were evaluated and reported along with the C-to-T percentage. As previous works on this topic documented that a significant change in *SLC6A4* methylation from birth to discharge was detected at CpG2 and CpG5 (47, 48), these two CpG sites were selected for the present study purposes.

#### Infants’ Socio-Emotional Response during the FFSF Paradigm

Infants’ negative emotionality was micro-analytically coded second-by-second as withdrawn, protesting, or displaying negative facial expressions (e.g., distress, cry face, grimacing), complaining, being fussy, and crying. A proportion index of negative emotionality was obtained dividing the total negative emotionality displayed during each FFSF by the actual length of the episode. Coders blind to group conditions were trained with a gold standard sample of 10 videotapes (percentage agreement = 83%).

#### Maternal Sensitivity

Maternal sensitivity was measured during the first face-to-face interaction (Play episode) of the FFSF paradigm according to the Global Rating Scales second Edition (GRS) by Murray and colleagues (unpublished).^1^ Maternal sensitivity is defined as a summary measure of warm, accepting, responsive and non-demanding caregiving behavior, and it is meant to measure how sensitively the mother responds to her infant signals in terms of how aware she is of even very subtle infant signals and willingness or reluctance to interact; how she empathizes and identifies with the infant and understands correctly what response is needed at a particular moment; and how responsive she is to the infant’s signals; and how appropriate her responses are. This system has documented discriminant validity with populations of infants at low- and high-risk, as well as cross-cultural validation (60, 61). Maternal sensitivity is coded on a global rating scale ranging from 1 (the mother does not try to interpret the majority of infant’s signals, she doesn’t respect his/her attempts to communicate and respond appropriately to his/her intentions; moreover, she may mock and laugh at him, with little or no sympathy at all) to 5 (the mother is highly sensitive for all the duration of the face-to-face interaction, producing frequent exaggeration, mirroring, and affirming responses to the infant’s signals). The Play episode of each dyad was coded for maternal sensitivity by two coders. They were masked to group membership and epigenetic data and percentage agreement was 90%.

#### Maternal Emotional State

As mothers of VPT infants might have greater depression and/or anxiety symptoms during the first months of life in comparison to FT counterpart (62), we measured both depressive and anxious symptoms in the two groups of mothers. Maternal depression was evaluated with the 21-item self-reported Beck Depression Inventory (BDI) (63). Items are rated on 4-point scales, indicating the absence/presence and severity of self-reported depressed feelings, behaviors, and symptoms. Maternal anxiety symptomatology was assessed by the STAI-Y (State-Trait Anxiety Inventory-form Y), 25 which is a 40-item Likert scale that measures the severity of state anxiety (items 1–20) as well as trait anxiety (items 21–40). Each item is rated on a 4-point intensity scale. Range of scores for each subtest is 20–80 with the higher score indicating greater anxiety.

### Plan of Analysis

Very preterm and FT infants were compared for neonatal and sociodemographic variables by means of *t*-test and *chi*^2^ test for continuous and categorical variables, respectively. Socio-emotional stress response was compared between VPT and FT infants through mixed model analysis of variance, with negative emotionality as the output variable and FFSF episodes as the within-subject factor (5 levels: Play, Stil-Face#1, Reunion#1, Still-Face#2, Reunion#2) and Group as the between-subject factor (2 levels: VPT vs. FT). Maternal sensitivity during the Play episode was compared between the two groups. To assess the moderating role of maternal sensitivity, two sets of stepwise multiple linear regressions were performed separately for VPT and FT infants.

Separate regression models are generally used when comparing preterm vs. FT infants’ outcomes [e.g., Ref. (49)] because VPT and FT infants’ life experience is very different and different confounders need to be controlled for. To select predictors, preliminary bivariate correlations were run to test the association between (a) maternal sensitivity and infants’ negative emotionality across the FFSF episodes; (b) *SLC6A4* CpG2 and CpG5 methylation and infants’ negative emotionality across the FFSF episodes. The final model was tested on infants’ negative emotionality considering only the FFSF episode(s) for which significant preliminary had emerged, and it included the following predictors: methylation of CpG sites significantly correlated with infants’ negative emotionality during the specific episode, maternal sensitivity during Play, and the interaction between CpG-specific *SLC6A4* methylation and maternal sensitivity. Finally, the regression model was controlled for clinical confounders. For both VPT and FT infants, confounders were: gestational age (weeks), maternal age (years), maternal educational level (years of study), family SES, maternal depression, maternal anxiety. Moreover, for VPT infants only, pain-related stress index, days on ventilation, and length of stay in the NICU was added as confounders. All the analyses were carried out using IBM SPSS Statistics 21, at α < 5%.

## Results

### Preliminary Results

Descriptive statistics for the sample are reported in Table 1. Gender distribution did not differ significantly between VPT and FT groups, *X^2^* = 0.11, *p* = 0.73.

**Table 1:** Descriptive statistics for the present sample.

	VPT infants (*n* = 27, 14 females)		FT infants (*n* = 26, 14 females)		
	Mean	SD	Mean	SD	*t*
**Neonatal variables**					
Gestational age (weeks)	31.07	1.73	39.64	1.22	−16.55***
Birth weight (grams)	1,512.04	336.13	3,372.31	402.49	−19.99***
Apgar at minute 1	6.48	1.40	9.77	0.69	−11.15***
**NICU-related variables**					
Length of NICU stay (days)	39.19	16.53	n.a.	n.a.	n.a.
Days on ventilation	12.50	13.98	n.a.	n.a.	n.a.
Pain-related stress index	13.00	14.11	n.a.	n.a.	n.a.
**Sociodemographic variables**					
Maternal age (years)	36.34	4.79	33.91	3.89	2.11*
Maternal education (years of study)	15.56	2.41	15.00	3.79	1.14
Family SES	57.69	17.28	65.96	20.30	−0.99
**Maternal psychological symptoms variables**					
BDI score	7.20	4.59	7.50	4.86	−0.60
STAI state score	29.64	6.71	30.75	6.65	−0.19
STAI trait score	35.50	6.14	36.45	6.11	−0.28

*VPT, very preterm; FT, full-term; NICU, Neonatal Intensive Care Unit; SES, socioeconomic status assessed according to Hollingshead (57); BDI, Beck Depression Inventory; STAI, State-Trait Anxiety Inventory*.

*p < 0.05, ***p < 0.001.

**Figure 2:**
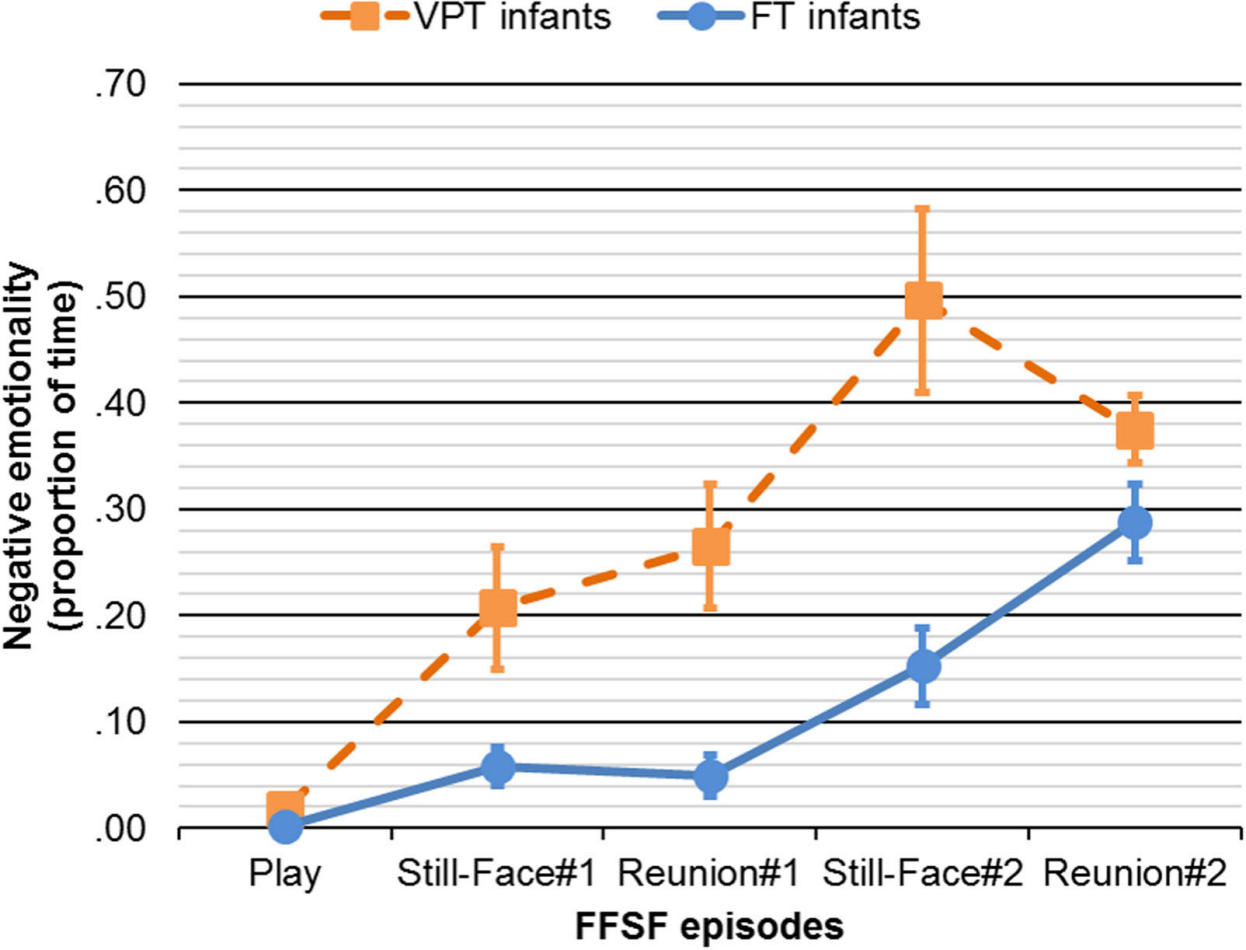
Negative emotionality through the FFSF episodes in VPT and FT infants. Continuous line represents FT infants. Dotted line represents VPT infants. FFSF, Face-to-Face Still-Face; VPT, very preterm; FT, full-term. Bars represent SEs.

Significant main effects of FFSF episodes, *F*(4, 48) = 20.89, η*^2^_p_* = 0.29, *p* < 0.001, and Group, *F*(1, 51) = 12.20, η*^2^_p_* = 0.19, *p* = 0.001, were better specified by the interaction effect FFSF episodes X Group, *F*(4, 48) = 4.34, η*^2^_p_* = 0.27, *p* = 0.004 (see Figure 2). Negative emotionality during Play did not differ between groups, *t*(51) = 1.90, *p* > 0.05. Compared to FT counterpart, VPT infants showed higher levels of negative emotionality during Still-Face#1, *t*(51) = 2.45, *p* = 0.018, Reunion#1, *t*(51) = 2.49, *p* = 0.018, Still-Face#2, *t*(51) = 3.51, *p* = 0.001, Reunion#2, *t*(51) = 3.65, *p* = 0.001.

### Preliminary Correlations

Preliminary correlations are reported in Table 2. No significant correlations emerged for both *SLC6A4* methylation and maternal sensitivity with infants’ negative emotionality during Still-Face#1 and Still-Face#2 as well as during Reunion#1. As such, only negative emotionality during Reunion#2 was tested in the regression model assessing the moderating role of maternal sensitivity on infants’ stress response. Moreover, as only *SLC6A4* methylation at CpG2 was found to be significantly correlated with infants’ stress negative emotionality, methylation at CpG5 was not included in the regression models.

**Table 2:** Preliminary correlations of *SLC6A4* CpG-specific methylation and maternal sensitivity with infants’ negative emotionality.

	VPT infants			FT infants		
Negative emotionality	CpG2 methylation	CpG5 methylation	Maternal sensitivity	CpG2 methylation	CpG5 methylation	Maternal sensitivity
Still-Face#1	0.08	0.14	−0.01	−0.02	−0.27	−0.24
Reunion#1	0.20	0.10	0.10	0.04	0.35	−0.23
Still-Face#2	0.43*	0.33	0.03	−0.14	0.09	−0.34
Reunion#2	0.40*	0.28	−0.01	0.25	0.18	−0.45**

*VPT, very preterm; FT, full-term*.

r, Pearson’s bivariate correlation coefficient are reported with levels of significance: *p < 0.05, **p < 0.01.

### Maternal Sensitivity Buffering Effect of Infants’ Socio-Emotional Stress Response

The regression model for VPT infants was significant, *R*^2^ = 0.12, *p* = 0.04. The only significant predictor of VPT infants’ negative emotionality during Reunion#2 was CpG2 methylation at VPTs’ discharge, *B* = 0.27, β = 0.40, 95% CI (0.01; 0.52), *t* = 2.15, *p* = 0.04. Higher CpG2 methylation at VPT infants’ discharge from the NICU was predictive of heightened negative emotionality during Reunion #2. When the model for VPT infants was controlled for birth weight as a confounder, the significant effect of CpG2 methylation on infants’ socio-emotional stress response was maintained.

**Figure 3:**
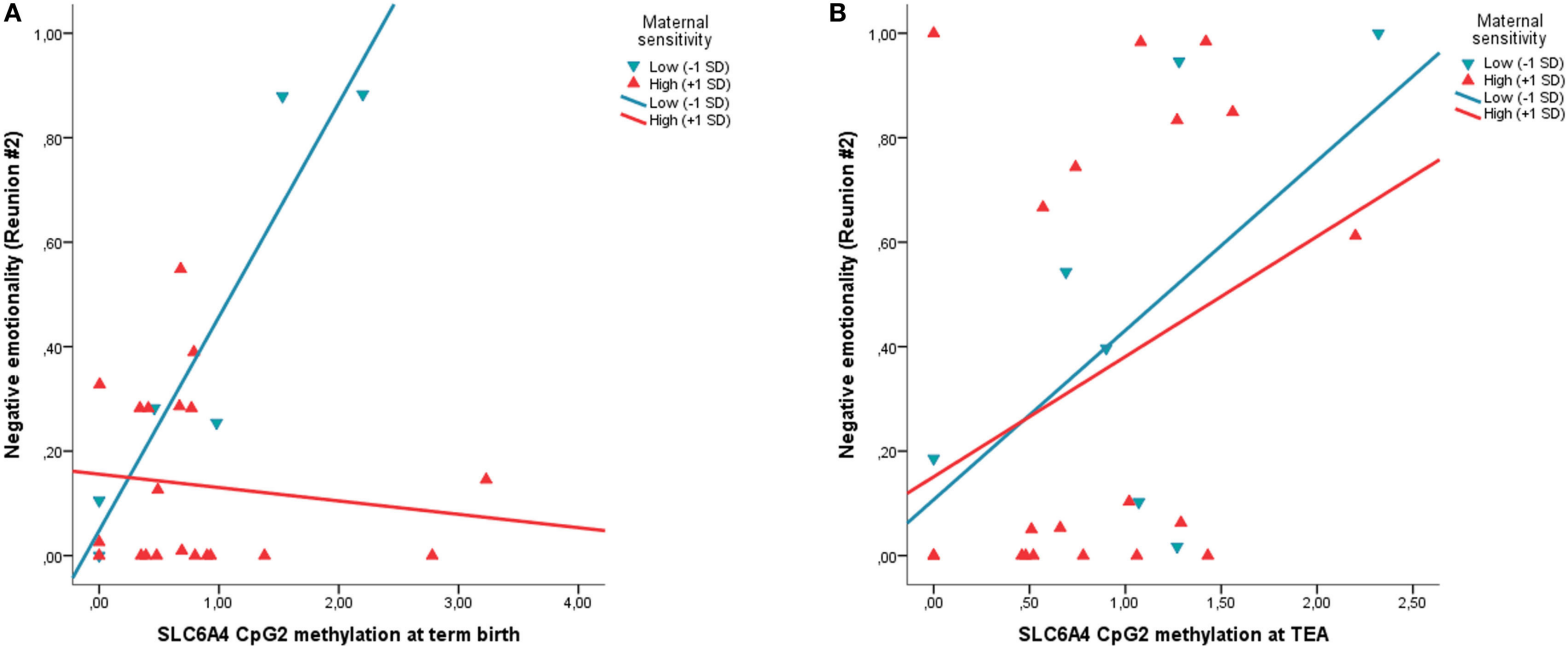
The moderating role of maternal sensitivity is observed on the association between *SLC6A4* CpG-specific methylation at birth in FT infants **(A)**, but not on the association between *SLC6A4* CpG-specific methylation at discharge in VPT infants **(B)**. Circles and dotted lines represent infants from low-sensitive mothers (−1 SD). Squares and continuous lines represent infants from high-sensitive mothers (+1 SD). Negative emotionality is measured as the proportion of time on the entire Reunion #2 episode; *SLC6A4*, serotonin transporter gene; CpG2, chr17: 28562786-28562787, −72 pb from the TSS; FT, full-term; VPT, very preterm.

The regression model for FT infants was significant, *R*^2^ = 0.31, *p* = 0.002. No significant effect of CpG2 methylation at FTs’ birth emerged, whereas maternal sensitivity significantly associates with FT infants’ negative emotionality during Reunion #2, *B* = −0.29, β = −0.49, 95% CI (−0.46; −0.12), *t* = −3.54, *p* = 0.002. Higher maternal sensitivity during the Play episode was predictive of less negative emotionality during Reunion #2 in FT infants. Moreover, a significant interaction effect between CpG2 methylation and maternal sensitivity emerged for the FT group, *B* = −0.43, β = −0.61, 95% CI (−0.65; −0.24), *t* = −4.43, *p* < 0.001. FT infants from mothers characterized by low maternal sensitivity showed a significant positive association between *SLC6A4* methylation of CpG2 and negative emotionality during Reunion #2, whereas no significant association emerged for FT infants from mothers with high maternal sensitivity (Figure 3). When the model was controlled for confounders, both the independent and interaction terms remained significant.

## Discussion

The aim of the present study was to assess the buffering role of maternal sensitivity on the association between *SLC6A4* methylation and VPT infants’ stress response compared to FT counterpart at 3 months CA. Preliminarily, we showed that during the FFSF procedure, VPT infants exhibited higher negative emotionality compared to FT infants, confirming previous evidence of the association between general poorer socio-emotional stress response and greater susceptibility to maternal unresponsiveness and prematurity (35, 38). Moreover, consistent with recent evidence (48), *SLC6A4* methylation at NICU discharge predicted greater negative emotionality during the repeated socio-emotional stress in VPT infants.

The present findings only partially confirmed our hypothesis that maternal sensitivity would reduce the effects of *SLC6A4* methylation on infants’ stress response, regardless of birth status (VPT vs. FT). Indeed, maternal sensitivity moderated the association between epigenetic status (i.e., methylation of the *SLC6A4* promoter region) and infants’ socio-emotional stress response in FT infants. Within the FT group, infants whose mothers showed high levels of sensitivity appeared to be protected from heightened negative emotionality in the face of increased levels of *SLC6A4* methylation, whereas a positive significant association was highlighted in infants of mothers characterized by low interactive sensitivity. This result is consistent with previous literature highlighting the buffering role of caregiver behavior in sustaining the development of adaptive socio-emotional stress response during early infancy (55, 64). Recently, Conradt et al. (7) showed that 4-month-old FT infants of depressed mothers characterized by high levels of interactive sensitivity had lower levels of CpG-specific methylation at a stress-related gene (i.e., the *NR3C1*) compared to infants of depressed mothers rated low in maternal sensitivity. The present study extends previous evidence to a different stress-related gene (i.e., *SLC6A4*), which is widely recognized for its role in socio-emotional development and regulation (13). Notably, the buffering effect of maternal sensitivity emerged only for the final Reunion episode of the FFSF procedure. This specificity might be better framed within the framework of the resilience hypothesis (65). According to this hypothesis, during the first months of life, a single and isolated exposure to maternal unresponsiveness (i.e., first Still-Face episode) is easily coped with by the infant and repaired by dyadic reunion and re-engagement. However, repeated exposures to maternal unresponsiveness (i.e., the second Still-Face episode) constitute a more robust and critical stressful exposure for infants and the capacity to regulate cumulative stress is much more challenged. Consistently, in the present sample, infants who had previously experienced higher maternal sensitivity in the face-to-face interaction (i.e., Play episode) showed better regulation at the end of the entire stressful procedure, suggesting that they had developed better capacity to regulate their behavior even under repeated-stress condition.

Contrary to our expectation, maternal sensitivity did not moderate the methylation-regulation association in VPT infants. Indeed, no association emerged between maternal sensitivity and infant negative emotionality. Importantly, mothers of VPT and FT infants did not differ in sensitivity level. Thus, the lack of maternal buffering in the VPT group might not be ascribed to reduced quality of maternal sensitivity. Rather, this finding might indicate that, in the VPT, the normal range of variability observed in maternal sensitivity during face-to-face interactions was not sufficient to support infant’s capacities to regulate socio-emotional stress. It should be noted that the early NICU-related stress exposure (e.g., pain-related stress) can have an adverse influence on infant’s stress response with long-term “programming” effects on future stress exposures (3). Indeed, a pattern of heightened stress susceptibility is generally observed in preterm infants during the first months of life (38–40, 54). Recent evidence further suggests that pain-related stress exposure during NICU is able to alter the main neuroendocrine system of stress response, i.e., the hypothalamic–pituitary–adrenal axis, resulting in a hyporesponsive activity under socio-emotional stress conditions at 3 months (58). As such, the neuroendocrine dysregulation of the stress response system might reduce the capacity to regulate behavior in response to repeated stress exposures (66). Consequently, we speculate that normally occurring variations in maternal sensitivity may be not enough to contrast the early stress programming due to adverse experiences and altered parental caregiving associated with NICU stay.

Consistent with previous studies (47, 67), we focused the epigenetic analyses on two specific CpG sites within the *SLC6A4* promoter region: the CpG 2 (Chr17: 28562786-28562787) and the CpG5 (Chr17: 28562847-28562848). Increased methylation in these sites has been previously associated with high levels of pain-related stress during NICU stay (both sites) (47), as well as with adverse developmental outcomes, including difficult temperament (CpG5) (53) and socio-emotional stress response (CpG2) (48). Here, we confirm previous findings about the role of CpG2 in infant’s socio-emotional stress response. In order to better frame the biological plausibility of this CpG site, it should be noted that this site is associated with H3K27Ac Mark often found near regulatory elements in genes’ promoter regions and has DNAse I hypersensitivity peak as detected in UCSC genome browser on human genome. Moreover, it has been previously associated to amygdala functional reactivity to negative emotional stimuli in human adults (67). Although the functional consequences of increased methylation at one specific CpG site still needs to be tested in RNA expression studies on VPT infants, it might be speculated that epigenetic alterations at this locus might be relevant for the regulation of serotonin transporter availability.

We acknowledge that the present study has limitations. First, the sample size was relatively small. Despite statistical power appeared to be enough to detect significant differences in DNA methylation at specific CpG sites, limits to the testing of additional contributing factors (e.g., infants’ gender) existed. Second, contrary to animal studies, behavioral epigenetic research in humans cannot rely on tissues from the central nervous system. We used two different peripheral tissues (i.e., cord blood and peripheral blood), consistent with previous research in the field (24). Although *SLC6A4* methylation has been measured from different tissues at birth and discharge, recent findings support the notion that cord blood methylation is maintained in peripheral blood methylation at mid-childhood (68). Moreover, Wang et al. (69) documented that *SLC6A4* DNA methylation obtained in peripheral tissues (i.e., peripheral blood) of this gene associates with *in vivo* measures of serotonin synthesis in children. Third, a three-way interaction with structural genetic variations of the *SLC6A4* (e.g., 5-HTTLPR polymorphism) was not plausible within the present sample. A recent review of the literature suggested that, even if just preliminary, targeting the interaction of polymorphic and epigenetic variations should be the more reliable approach to assess the effects of variable transcriptional activity of the *SLC6A4* gene on behavioral and physiological outcomes (21). Thus, caution is suggested when interpreting the present findings, as it is likely that multiple genetic mechanisms may contribute to behavioral development and temperament. Fourth, maternal sensitivity is meant to be a multidimensional construct (70, 71), including responsive parenting, contingent responses to infants’ cues, non-intrusive and non-demanding maternal behaviors. Hence, future studies should target the epigenetic effects of specific maternal behaviors on infants’ stress response development, also including other stress-related genes [e.g., *BDNF* (72); *NR3C1* (73)] mimicking what has been done in behavioral epigenetic research on animal models (74). For instance, as proposed by Conradt et al. (7), it is plausible to hypothesize that sensitive touch by the mother might be a potential proxy for environmental-driven epigenetic regulation of infants’ stress-related genes and recent retrospective research appears to confirm such regulatory role of maternal touch in healthy adults (8).

## Conclusion

In the present study, maternal sensitivity emerged as a protective factor buffering the early effects of *SLC6A4* epigenetic variations on stress-response capacities in 3-month-old FT infants, but not in VPT infants. The lack of a sensitivity buffering effect in the VPT group is somehow surprising. Notwithstanding, it may provide new insights about the conditions by which the quality of early caregiving supports VPT infants’ socio-emotional development and protect them against the risk of long-lasting programming of altered stress response. It should be noted that VPT infants and their mothers are precociously separated after birth (75). Indeed, it might be speculated that the buffering effect of maternal sensitivity observed in FT infants was disrupted by the altered early mother–infant contact due to NICU stay in the VPT group. Notably, the very first hours and days after VPT birth appear to be a sensitive period to help VPT infants and their mothers develop an intimate physical and emotional bond through skin-to-skin contact (76). As such, the present study indirectly suggests that specific early interventions might be needed in order to observe an effective buffering effect of maternal sensitivity on the association between precocious adverse-related epigenetic alterations (i.e., *SLC6A4* methylation) and the subsequent socio-emotional stress response of VPT infants. NICU developmental care interventions (e.g., skin-to-skin contact, maternal holding) during the NICU stay are known to promote mother–infant closeness after VPT birth and to balance the effects of NICU-related stress on VPT infants’ behavioral and physiological stress response (77).

Taken together, these evidences support the hypothesis that the effects of maternal sensitivity might be time dependent and that the critical period for a buffering effect on VPT infants socio-emotional stress response might be altered. In animal models, the seminal work by Meaney and colleagues (78, 79) documented that the timing of early exposure to high-quality maternal sensitivity is critical in reverse epigenetic alterations due to early adverse events and to protect the long-lasting programming in the offspring. As such, we suggest that future research should focus on the epigenetic correlates of early mother–infant interventions in the NICU. Such studies hold the potentials to prove the hypothesis that providing precocious support in a sensitive period of development might counter-balance, if not reverse, the methylation effects of early adversities on VPT infants’ development.

## Author Contributions

LP contributed to study conception and design, acquisition, analysis, and interpretation of data, drafting of the manuscript. MF contributed to data acquisition, interpretation of findings, final approval of manuscript. RG and UP contributed to data analysis. FMorandi and IS contributed to data acquisition. FMosca and RB contributed to study conception and approved the final version of the manuscript. RM contributed to study conception and design, interpretation of findings, drafting of the manuscript, and approval of the final version.

## Conflict of Interest Statement

The authors declare that the research was conducted in the absence of any commercial or financial relationships that could be construed as a potential conflict of interest.

## Footnotes

^1^Fiori-Cowley A, Murray L, Gunning M. Global Rating Scales of Mother-Infant Interaction at Two and Four Months. 2nd ed. (2000).
